# Indole-3-carbinol (I3C) reduces apoptosis and improves neurological function after cerebral ischemia–reperfusion injury by modulating microglia inflammation

**DOI:** 10.1038/s41598-024-53636-6

**Published:** 2024-02-07

**Authors:** Long Peng, Xingjia Zhu, Chenxing Wang, Qiaoji Jiang, Shian Yu, Gaochao Song, Qianqian Liu, Peipei Gong

**Affiliations:** 1 Department of Neurosurgery, Affiliated Hospital of Nantong University, Medical School of Nantong University, Nantong, China; 2 Department of Neurosurgery, Ganzhou Hospital of Guangdong Provincial People’s Hospital, Ganzhou Municipal Hospital, Ganzhou, China; 3 Department of Neurosurgery, Affiliated Yancheng Clinical College of Xuzhou Medical University, Yancheng, 224000 Jiangsu Province China; 4 Department of Surgery, Infectious Disease Hospital Affiliated to Nanchang University (Nanchang Ninth Hospital), Nanchang, China; 5 Department of Neurosurgery, The Second Affiliated Hospital of Nanjing Medical University, Nanjing, China

**Keywords:** Drug discovery, Neuroscience, Diseases, Neurology

## Abstract

Indole-3-carbinol(I3C) is a tumor chemopreventive substance that can be extracted from cruciferous vegetables. Indole-3-carbinol (I3C) has been shown to have antioxidant and anti-inflammatory effects. In this study, we investigated the cerebral protective effects of I3C in an in vivo rats model of middle cerebral artery occlusion (MCAO). 8–10 Week-Old male SD rat received I3C (150 mg/kg, once daily) for 3 days and underwent 3 h of middle cerebral artery occlusion (MCAO) followed by reperfusion. The results showed that I3C pretreatment (150 mg/kg, once daily) prevented CIRI-induced cerebral infarction in rats. I3C pretreatment also decreased the mRNA expression levels of several apoptotic proteins, including Bax, caspase-3 and caspase-9, by increasing the mRNA expression levels of the anti-apoptotic protein Bcl-2. Inhibited apoptosis in the brain cells of MCAO rats. In addition, we found that I3C pretreatment reduced neuronal loss, promoted neurological recovery after ischemia–reperfusion injury and increased seven-day survival in MCAO rats. I3C pretreatment also significantly reduced the expression of inducible nitric oxide synthase (INOS), interleukin-1β (IL-1β) and interleukin-6 (IL-6) mRNA in ischemic brain tissue; Increased expression of interleukin-4 (IL-4) and interleukin-10 (IL-10) mRNA. At the same time, I3C pretreatment significantly decreased the expression of the M1 microglial marker IBA1 after cerebral ischemia–reperfusion injury and increased the expression of these results in the M2 microglial marker CD206. I3C pretreatment also significantly decreased apoptosis and death of HAPI microglial cells after hypoxia induction, decreased interleukin-1β (IL-1β) and interleukin-6 (IL-6) mRNA The expression of interleukin-4 (IL-4) and interleukin-10 (IL-10) mRNAs was increased. These results suggest that I3C protects the brain from CIRI by regulating the anti-inflammatory and anti-apoptotic effects of microglia.

## Introduction

The second leading cause of death worldwide is stroke, which accounts for 11.13 percent of all deaths. Ischemic strokes cause 87% of stroke-related deaths. Early systemic thrombolytic therapy has been shown to be the most effective treatment for ischemic stroke and significantly reduced mortality^1^. However, the process of recovery of the ischemic area can cause secondary damage and lead to irreversible death of nerve cells, called cerebral ischemia–reperfusion injury(CIRI)^2^. Although endovascular thrombectomy (EVT) is highly effective, this treatment is only indicated for large vessel lesions and relies on excellent technique and skilled specialists^3^. There are no standard clinically applicable therapy for treating and/or preventing CIRI. Therefore, it is clinically important to explore new ways to prevent and treat CIRI.

The process of acute ischemia–reperfusion injury is thought to be mediated by the inflammatory response^4^. Scientific study shows that intake of cruciferous vegetables significantly reduces risk of stroke^5^^.^ That may be related to the natural ingredients in it. Indole-3-carbinol (I3C) is a promising phytochemical found abundantly in cruciferous vegetables, such as broccoli and cauliflower^6^. I3C is generated when these plants are cooked, cut or chewed via the myrosinase-catalyzed hydrolysis of glucobrassicin, an indole glucosinolate^7^. Scientific studies have shown that I3C has powerful pharmacological effects, including anti-inflammatory, antioxidant, antiviral, antithrombotic activity and anti-cancer^8–11^. I3C was found to be effective against ischemic reperfusion injury of striated muscles by reducing the transcriptional activity of NFkB^12^. In a mouse model of high-fat diet (HFD), I3C supplementation was found to inhibit the high-fat diet-induced increase in mRNA expression levels of pro-inflammatory factors such as tumor necrosis TNF-α, IL-6, and interferon β^13^. I3C exerts its anti-tumor effects by affecting the Akt/NF-κB signaling network, which regulates signaling pathways for cell cycle progression, survival and metastasis^14^. I3C can also suppresses chronic inflammation-driven mouse lung tumorigenesis^15^. Recently, I3C treatment has been reported to attenuate oxidative stress indicators such as dihydroethidium (DHE), malondialdehyde (MDA), reactive oxygen species (ROS), nitric oxide (NO) and inflammatory indicators such as tumor necrosis factor α, interleukin 1α and interleukin 6β after cardiac ischemia–reperfusion injury, thus providing cardioprotective effects^16^.

I3C treatment have protective effect on neurological function after cerebral ischemia and prevents thrombosis by inhibiting platelet aggregation^17^. In addition, I3C also regulates microglia intracellular homeostasis when microglia challenged with inflammatory stimuli^18^. Scientific study shows that M2 microglia reduce inflammatory stimulation of reperfusion injury after ischemic stroke^19^. However, to date, the direct effect of I3C treatment on ischemia–reperfusion-induced cerebral injury is unclear. In the present study, we investigated this issue using the MCAO model in SD rats and the oxygen–glucose deprivation/reoxygenation (OGD/R) model in HAPI cells. We investigated the effects of I3C treatment on microglia regulation, apoptosis and pro-inflammatory responses in ischemic brain tissue stimulated rats and also investigated the effects of I3C addition on HAPI microglia under oxygen–glucose deprivation stimulation in vitro.

## Results

### Protective effect of I3C on ischemia–reperfusion injury and neurological function in rat brain

**Figure 1:**
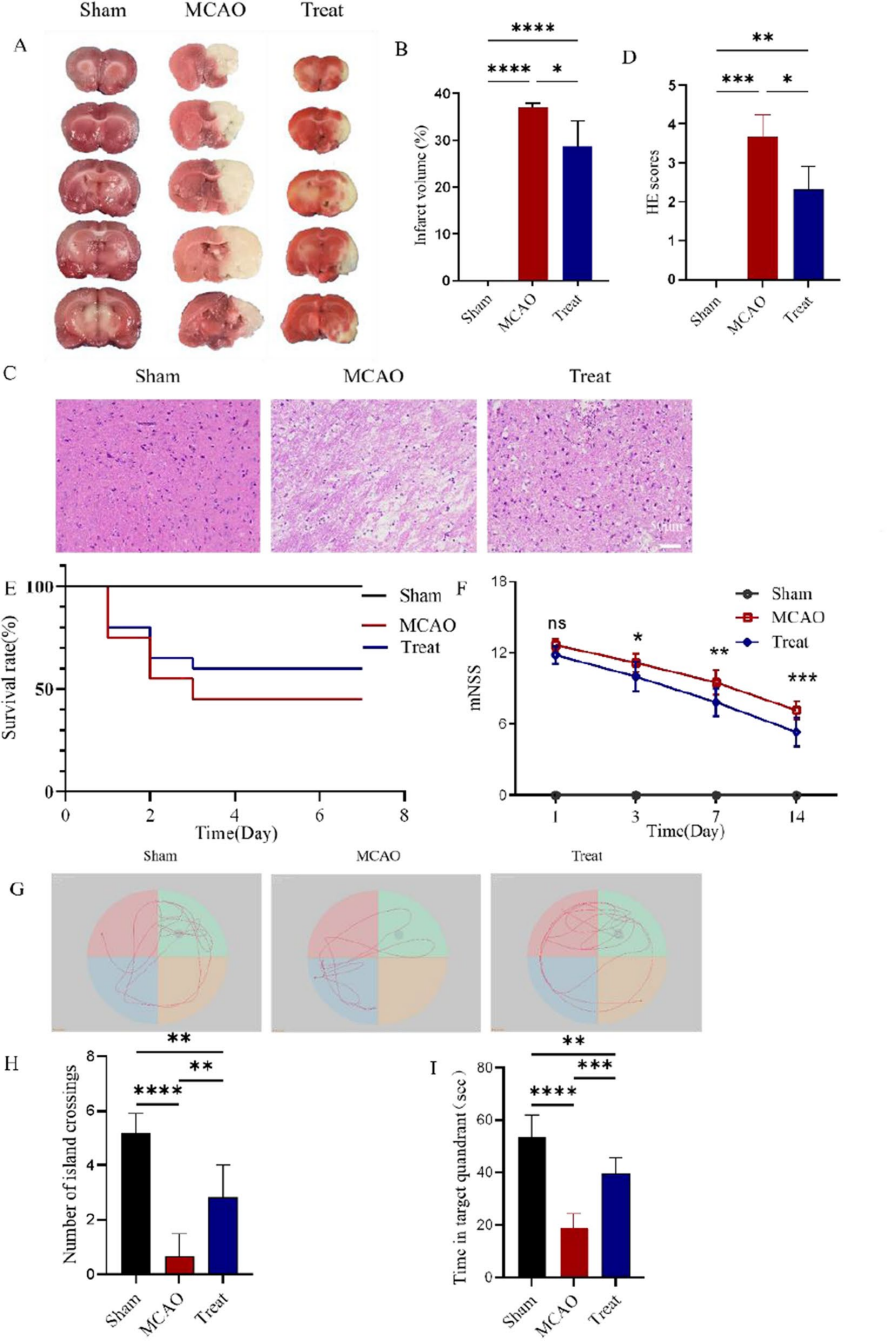
Effect of I3C on Ischemia/reperfusion -induced brain tissue damage and neurological function. (**A**) representative photograph showing the TTC staining of brain of rats with or without treatment with I3C (150 mg/kg). The non-ischaemic area is red and the infarcted area is pale white. (**B**) Quantitative analysis showing the size of brain infarction area in rats treated with or without I3C 3 h after reperfusion. (**C**) Representative hematoxylin–eosin (HE) staining results showing that I3C pretreatment reduced Ischemia/reperfusion -induced brain tissue damage in rats. (**D**) Quantitative analysis showing brain tissue damage in rats treated with or without I3C after 3 h of reperfusion. (**E**) Analysis of the seven-day survival rate of each group. (**F**) Quantitative analysis showing that I3C pretreatment improved Ischemia/reperfusion -induced mNSS scores (n = 6, *p < 0.001 vs. sham treatment, *p < 0.05 vs. Ischemia/reperfusion). (**G**) Representative results comparing the Morris water maze in the sham-operated group, MCAO rats, and rats after I3C treatment. (**H**) Quantitative statistics of the number of island penetrations in the sham-operated group, MCAO rats, and rats after I3C treatment. (**I**) Quantitative statistics of the time to reach the target quadrant in rats. Scale bar: 50 μm .Data are expressed as mean ± SEM. The p-values in Fig. 1F were analysed using two-way ANOVA, and the remaining p-values were analysed using one-way ANOVA. n.s.P ≥ 0.05,*P < 0.05,**P < 0.01,.***P < 0.001,****P < 0.0001.

Figure 1A shows a representative image of TTC staining, and Fig. 1B indicates the infarction volume of the rats in the figure. No cerebral infarction were seen in the brains of rats in the sham-operated group. Cerebral infarction volume in the MCAO group (37.21% ± 0.77%) was significantly lower in the I3C + MCAO group (29.67% ± 5.2%) than in the MCAO group (P < 0.05). The results of hematoxylin–eosin staining showed no obvious pathological changes in the sham-operated group rats, brain tissue damage in the MCAO group rats with disordered cell arrangement and irregular morphology, and significant improvement in the morphological structure of brain tissue in the I3C-treated group rats (Fig. 1C), suggesting that I3C pretreatment could reduce the brain tissue damage caused by ischemia–reperfusion. The H&E semi-quantitative score of brain tissue was significantly higher in the MCAO group than in the sham-operated group (P < 0.05). The H&E semi-quantitative score was significantly lower in the I3C-treated group than in the MCAO group (P < 0.05 Fig. 1D). The seven-day survival rate of rats in the post-surgical I3C supplementation group was significantly higher than that of the MCAO group (Fig. 1E). From day 1 to day 14 after surgery, the modified neurological severity score (mNSS) was significantly better in the I3C treatment group than in the MCAO group (P < 0.001 Fig. 1F). I3C-treated MCAO rats had a significantly better number of island penetrations and time to find the target quadrant in the water maze experiment than MACO rats (P < 0.01, P < 0.001 Fig. 1G–I).

### I3C inhibits neural cell apoptosis in ischemia–reperfusion injury in rat brain

**Figure 2:**
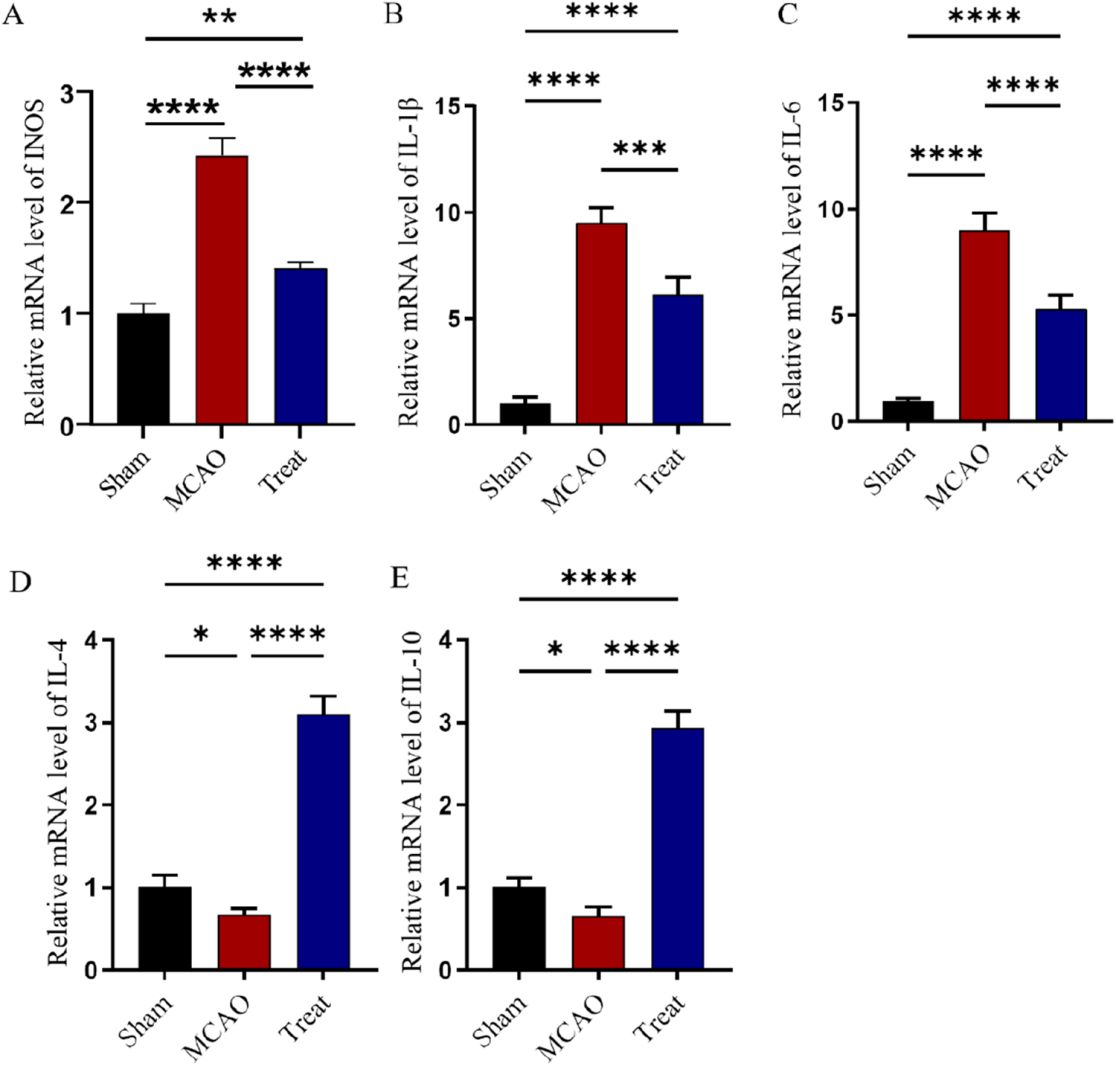
Effect of I3C on inflammatory parameters in brain tissue of Ischemia/reperfusion rats. (**A**–**C**) PCR analysis showed that I3C pretreatment (150 mg/kg) inhibited Ischemia/reperfusion -induced ischemic brain tissue in INOS (**A**) (n = 3, ****p < 0.0001 vsMCAO), IL-1β (**B**) (n = 3, ***p < 0. 001 vsMCAO) and IL-6 (n = 3, ****p < 0.0001 vsMCAO) (**C**) expression and enhanced the increased mRNA expression levels of IL-4 (**D**) and IL-10 (**E**) (n = 3, ****p < 0.0001 vsMCAO). Data are expressed as mean ± SEM. P-values were calculated using one-way· ANOVA. n.s.P ≥ 0.05,*P < 0.05,**P < 0.01,.***P < 0.001,****P < 0.0001.

**Figure 3:**
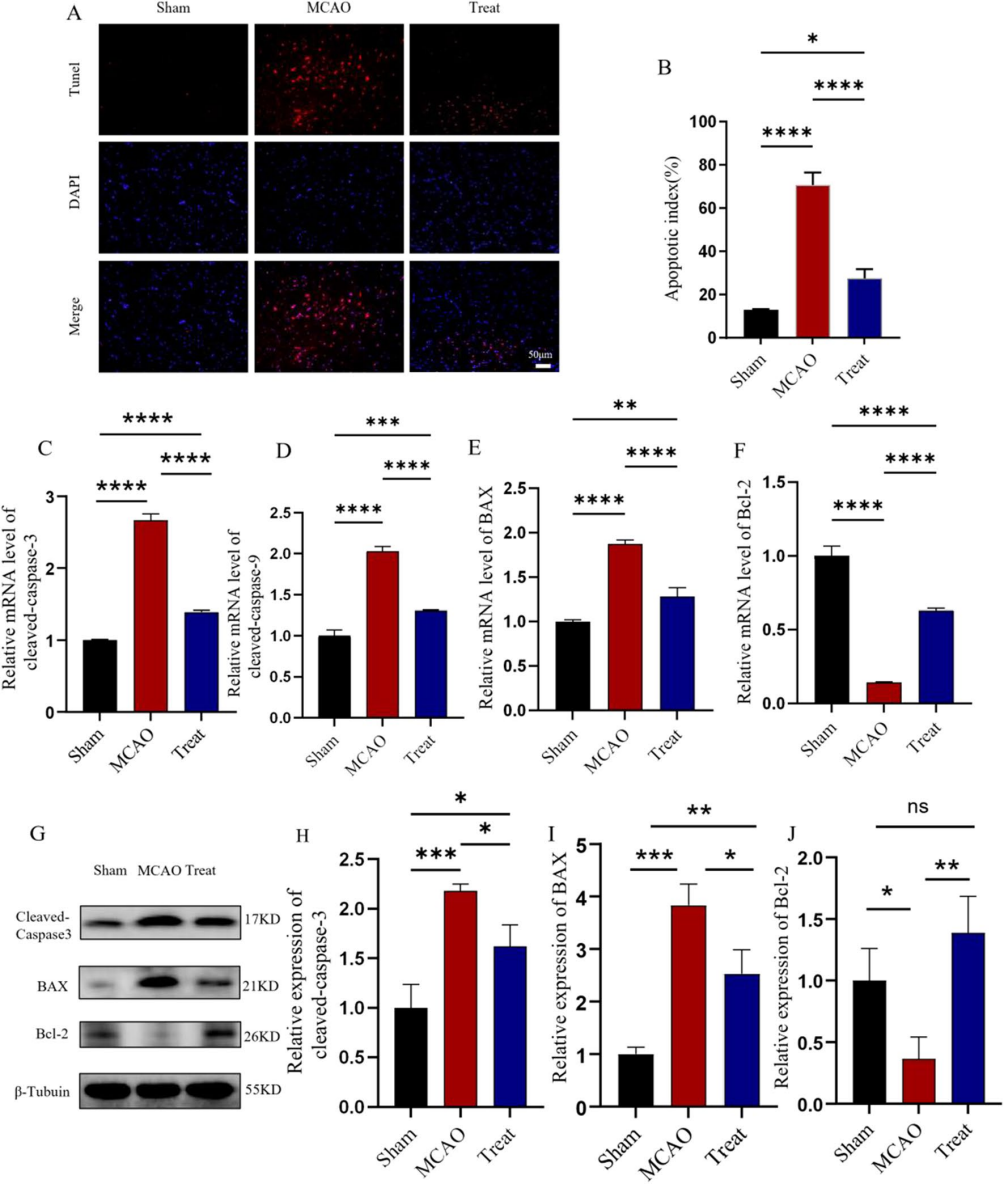
Effect of I3C on Ischemia/Reperfusion-induced apoptosis in neuronal cells. (**A**,**B**) I3C pretreatment prevented Ischemia/reperfusion -induced increase in the number of tunel-positive cells in ischemic brain tissue (n = 3, ***p < 0.001, compared with MCAO). (**C**,**E**) I3C pretreatment (150 mg/kg) prevented I/ r-induced increases in the expression levels of Bax (**C**), caspase-3 (**D**) and caspase-9 (**E**) in ischemic brain tissue (n = 3, ****p < 0.0001). I3C pretreatment prevented Ischemia/reperfusion -induced increases in the expression levels of BCL-2 (**F**) in ischemic brain tissue (n = 3, ****p < 0.0001). expression levels from being reduced (n = 3, ****p < 0.0001, compared with MCAO). (**G**) Representative protein blotting results of apoptosis-related protein expression in brain tissue from ischaemic regions of rats. (**H**) Quantitative analysis of caspase-3/β-Tubulin Western blot results. (**I**) Quantitative analysis of BAX/β-Tubulin Western blot results. (**J**) Quantitative analysis of Bcl-2/β-Tubulin Western blot results. Scale bar: 50 μm. Data are expressed as mean ± SEM. P-values were calculated using one-way· ANOVA. n.s.P ≥ 0.05,*P < 0.05,**P < 0.01,.***P < 0.001,****P < 0.0001.

**Figure 4:**
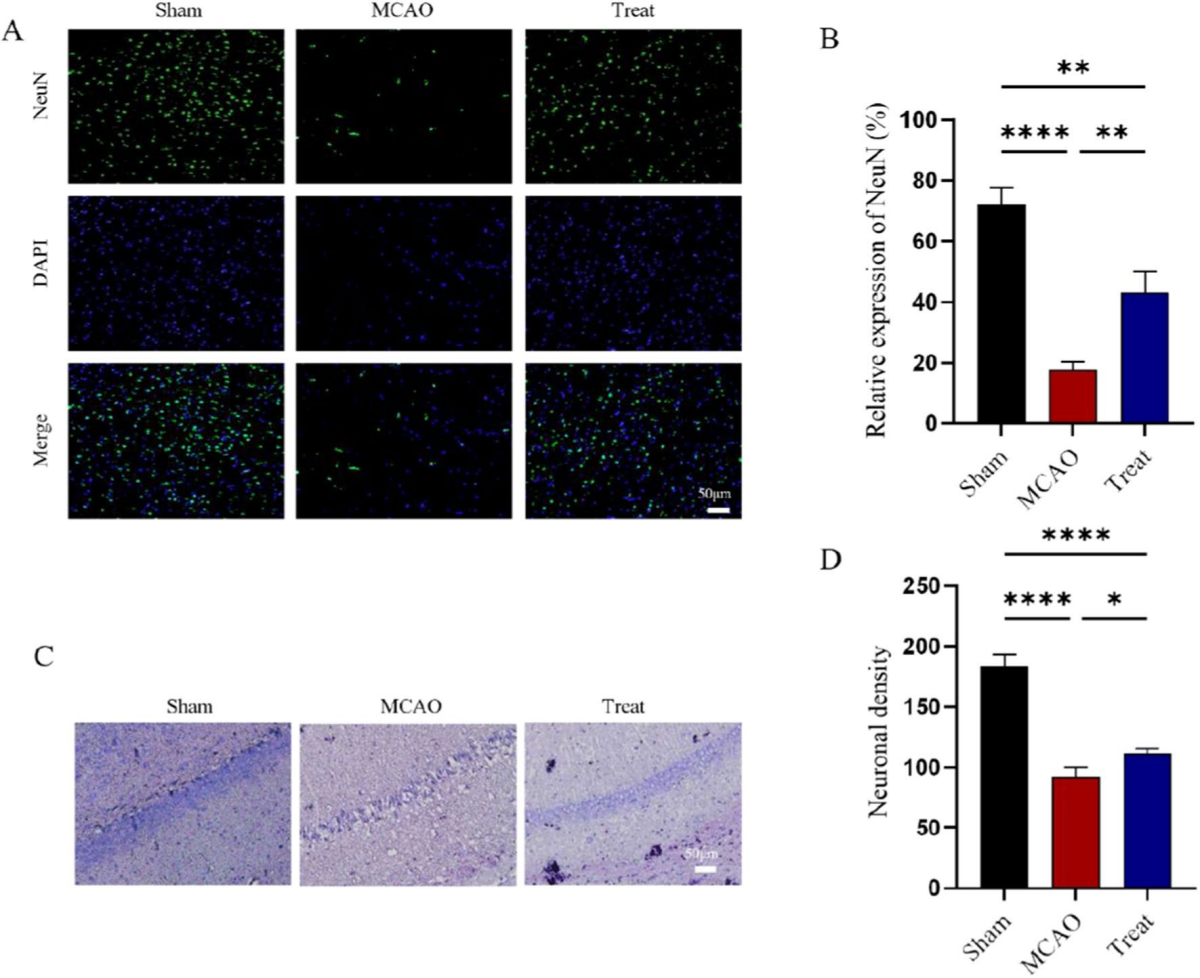
Effect of I3C on Ischemia/reperfusion -induced neuronal loss. (**A**,**B**) I3C pretreatment prevents Ischemia/reperfusion -induced neuronal loss (n = 3, **p < 0.01, compared with MCAO). (**C**,**D**) I3C pretreatment (150 mg/kg) prevented I/ r-induced hippocampal neuronal loss (n = 3, *p < 0.05, compared to MCAO). Scale bar: 50 μm. Data are expressed as mean ± SEM. P-values were calculated using one-way· ANOVA. n.s.P ≥ 0.05,*P < 0.05,**P < 0.01,.***P < 0.001,****P < 0.0001.

Next, we experimentally investigated the effect of I3C supplementation on apoptosis in brain tissue after MCAO/R-induced cerebral ischemia–reperfusion injury in rats (Fig. 2). Analysis of the TUNEL staining results revealed a significant increase in the number of TUNEL-positive cells in the brain tissue of MCAO/R rats after cerebral ischemia–reperfusion, compared with a significant decrease in the 3C-treated group (Fig. 3A,B, P < 0.0001). We also examined the changes in the expression of apoptosis-related proteins caspase-3, caspase-9, Bax and BCL-2 mRNA. Changes in the expression of caspase-3 and caspase-9 in the brain tissue of rats in the MCAO/R and I3C supplemented groups after ischemia–reperfusion (Fig. 3C,D). The results showed that the expression levels of three pro-apoptotic proteins, BAX (Fig. 3C, P < 0.0001), caspase-3 (Fig. 3D, P < 0.0001) and caspase-9 (Fig. 3E, P < 0.0001), were significantly decreased after I3C treatment, while the expression levels of the anti-apoptotic protein BCL-2 mRNA were significantly increased (Fig. 3F, P < 0.0001). We also examined the protein expression levels of caspase-3, BAX, and BCL-2 (Fig. 3G–J), and the results were consistent with the PCR results that the expression levels of two pro-apoptotic proteins, caspase-3 (Fig. 3G, P < 0.05) and BAX (Fig. 3H, P < 0.05), were significantly decreased after I3C treatment, and the expression level of the anti-apoptotic protein BCL-2 was significantly increased (Fig. 3J, P < 0.01).We also observed neuronal loss by immunofluorescence and Nissl staining, and the results showed that I3C treatment significantly reduced neuronal loss after ischemia–reperfusion injury (Fig. 4A,B, P < 0.01, and Fig. 4C,D, P < 0.05). These results suggest that I3C supplementation attenuates apoptosis and neuronal loss in MCAO/R-induced cerebral ischemia–reperfusion injury in rats.

### I3C inhibits inflammatory responses, apoptosis and cell death in the HAPI microglia OGD/R model

**Figure 5:**
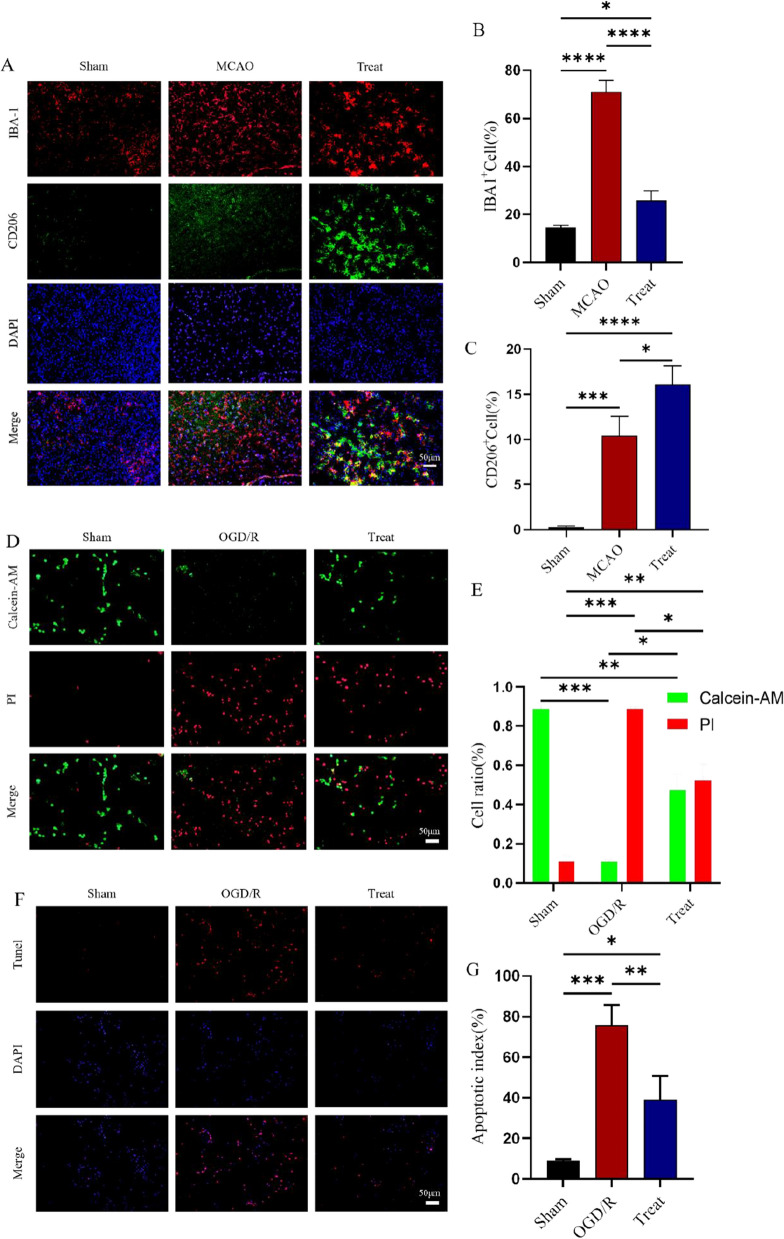
Effect of I3C on Ischemia/reperfusion -induced microglia. (**A**,**C**) I3C pretreatment (150 mg/kg) inhibits Ischemia/reperfusion -induced expression of the M1-type microglial marker IBA1 (n = 3, ****p < 0.0001, vs. MCAO) and promotes increased expression of the M2-type microglial marker CD206 (n = 3, *p < 0.05, vs. MCAO). (**D**,**G**). I3C Pretreatment reduced OGD/R-induced apoptosis and death of HAPI microglia (n = 3, **p < 0.01, compared to OGD/R). Scale bar: 50 μm. Data are expressed as mean ± SEM. The p-values in Figure E were analysed using two-way ANOVA, and the remaining p-values were analysed using one-way ANOVA. n.s.P ≥ 0.05,*P < 0.05,**P < 0.01,.***P < 0.001,****P < 0.0001.

**Figure 6:**
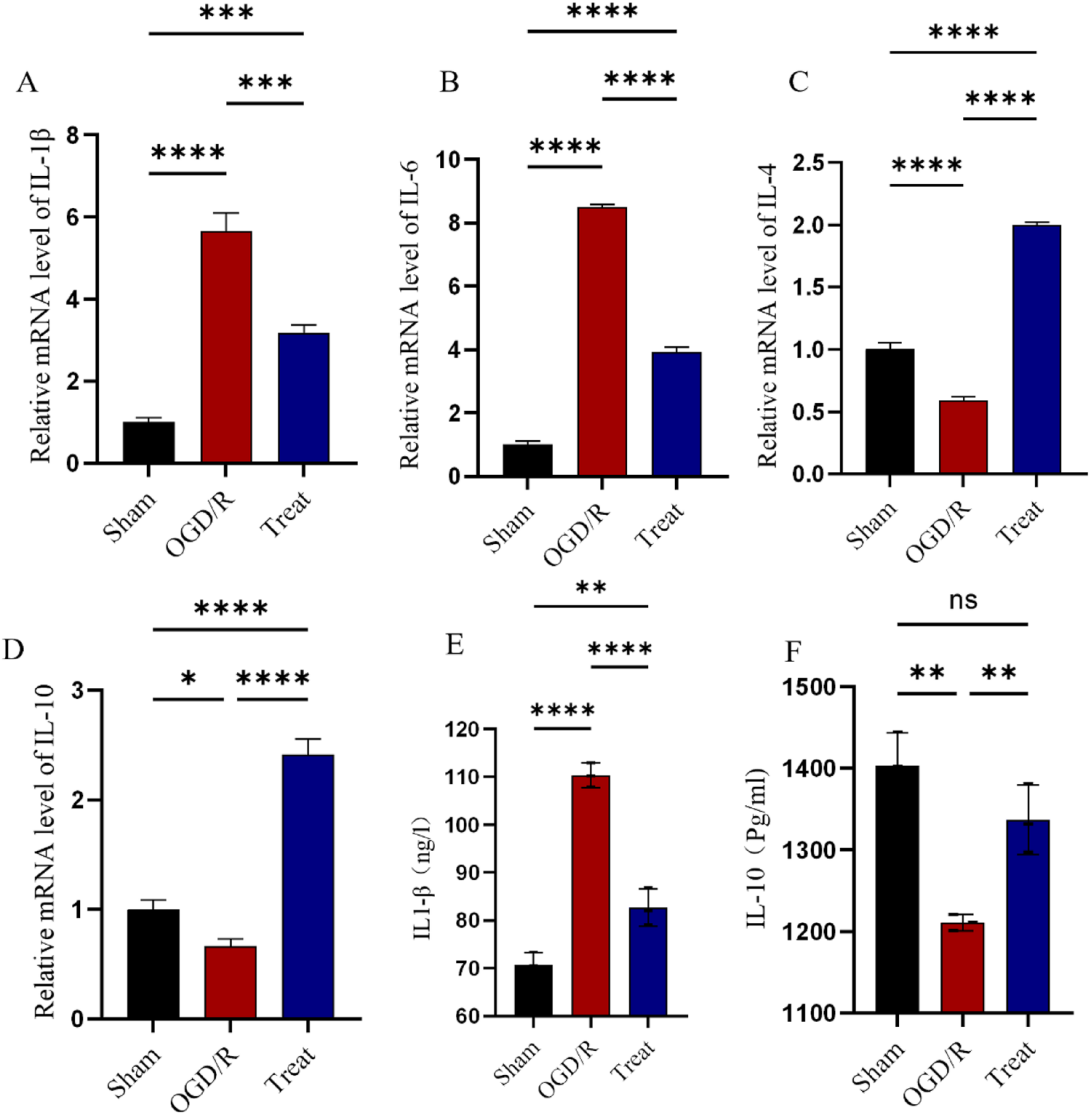
Effect of I3C on OGD/R-induced inflammatory parameters in HAPI microglia. PCR analysis revealed that (**A**,**B**) I3C pretreatment inhibited OGD/R-induced IL-1β (**A**) (n = 3, ***p < 0.001 vs OGD/R) and IL-6 (**B**) (n = 3, ****p < 0. 001 vs OGD/ R) expression and enhanced the increased mRNA expression levels of IL-4 (**C**) and IL-10 (**D**) (n = 3, ****p < 0.0001 vs OGD/R). Data are expressed as mean ± SEM. (**E**) ELISA results of IL1β expression levels in HAPI microglial cell culture medium. (**F**) ELISA results of IL10 expression levels in HAPI microglial cell culture medium. P-values were calculated using one-way· ANOVA. n.s. p ≥ 0.05,*p < 0.05,**p < 0.01,.***p < 0.001,****p < 0.0001.

Next, we examined indicators of apoptosis, death and inflammation in the HAPI microglia OGD/R model. The results showed that I3C pretreatment significantly reduced hypoxia-induced apoptosis (P < 0.01, Fig. 5F,G) and death (P < 0.05, Fig. 5D,E) and inhibited the expression of the pro-inflammatory factors IL-1β (Fig. 6A, P < 0.001, Fig. 6E, P < 0.001) and IL-6 (Fig. 6B, P < 0.0001) and increased the pro-inflammatory factors IL-4 and IL-10 expression (Fig. 6C,D, P < 0.0001, Fig. 6F, P < 0.001). In addition, CCK8 cell experiments showed massive cell death due to glyoxyl deprivation after OGD/R modelling, whereas I3C treatment significantly reduced cell death (Supplementary Figure S2, P < 0.01).

### Protective effect of I3C on cerebral ischemia–reperfusion-induced inflammatory response

As shown in Fig. 2A–E, the expression levels of INOS (P < 0.0001, Fig. 2A), IL-1β (P < 0.001, Fig. 2B) and IL-6 (P < 0.0001, Fig. 2C) were significantly increased and the expression levels of IL-4 (P < 0.0001, Fig. 2D) and IL-10 (P < 0.0001, Fig. 2E) were decreased in the MCAO group. We used immunofluorescence co-localisation to verify that the validation factor INOS co-localised with the microglia marker IBA1, demonstrating that the inflammatory factor originated from microglia (Supplementary Figure S1A, B). I3C The treatment group significantly decreased the mRNA expression of pro-inflammatory factors IL-1β (P < 0.001, Fig. 2B) and IL-6 (P < 0.0001, Fig. 2C), while increasing the inflammatory factors IL-4 (P < 0.0001, Fig. 2D) and IL-10 (P < 0.0001, Fig. 2E) in the ischemic brain tissue of rats. This result suggests that supplementation with I3C prevents the development of pro-inflammatory responses in ischemic brain tissue. Next, we tested IBA1, an indicator of activated microglia, and CD206, a marker of M2-type microglia (Fig. 5A), and showed that I3C treatment significantly decreased the number of activated microglia and increased the number of M2-type microglia (Fig. 5B,C), which are inhibitory to inflammation. This is further evidence that I3C may antagonise early inflammation after cerebral ischaemia/reperfusion injury by modulating microglia.

## Discussion

Cerebral ischemia can lead to brain injury, which is exacerbated by reperfusion, and is referred to as cerebral ischemia/reperfusion injury. The pathogenic mechanisms of cerebral ischemia–reperfusion injury are complex and result from a range of factors, including inflammatory responses, apoptosis^20,21^. Microglia activation when the brain microenvironment is stimulated by inflammation^22^. Microglia play a dual role in neuroinflammation. During cerebral ischemia–reperfusion injury, M2 microglia are the predominant phenotype and exert a protective effect on neuronal cells, whereas M1 microglia produce inflammatory mediators that are harmful to brain tissue^23^. However, during the course of cerebral ischemia–reperfusion disease M2 microglia gradually decrease^23^. In this study, we investigated the pharmacological effects and therapeutic mechanisms of I3C-regulated microglia on cerebral ischemia–reperfusion injury in rats using the MCAO/R model and the OGD/R model. We experimentally found that I3C treatment can was effective in reducing the cerebral infarction volume, protecting neurological function and reducing neuronal loss after cerebral ischemia–reperfusion injury in rats, and improving the 7-day survival rate after cerebral ischemia–reperfusion injury in rats. These results suggest that I3C may be effective in the treatment of cerebral ischemia–reperfusion injury and provide further evidence for the cerebral protective effect of I3C.

Ischemia–reperfusion stimulation is known to lead to a pro-inflammatory response and increased apoptosis of neuronal cells such as microglia in ischemic brain tissue^23,24^. However, it remains unclear exactly how I3C prevents cerebral ischaemia/reperfusion injury. AhR is a ligand-dependent transcription factor that can be activated by a variety of ligands, has immunomodulatory activity and is a potential target for I3C^18,25^. Upon binding to the ligand, it separates from the chaperone and disperses to the nucleus, where it maintains cellular homeostasis by regulating gene transcription^26^. One investigator found that pretreatment of hypoxia-induced H9c2 cells with I3C reduced the expression of TNF-α, IL-1β and IL-6 mRNA by activating the AhR pathway^27^. Data from real-time PCR experiments showed that I3C pretreatment effectively inhibited the expression of pro-inflammatory factors IL-1β and IL-6 mRNA in Ischemia/reperfusion-induced ischemic brain tissue, and simultaneously increased the expression of anti-inflammatory factors IL-4 and IL-10 mRNA, suggesting that I3C may have an anti-inflammatory effect onIschemia/reperfusion injury in the brain (Fig. 5). Immunofluorescence results showed increased expression of the M2 microgel marker CD206 after I3C treatment (Fig. 4).This finding is somewhat consistent with previous in vitro results: I3C pretreatment was found to reduce the increase in IL-1β and IL-6 mRNA expression in LPS-stimulated microglia^18^. I3C pretreatment also reduced hypoxia-induced HAPI cell death and apoptosis. This result, in turn, was somewhat similar to a previous study by^18^: I3C treatment promoted lps-induced improvement in microglia phagocytosis and migration. The present study speculates that I3C treatment may exert anti-inflammatory effects in the rat MCAO model through the modulation of microglia, pending further confirmation in future studies.

Our TUNEL assay showed that I3C pretreatment prevented Ischemia/reperfusion-induced apoptosis in ischemic brain tissue, which is consistent with caspase-3 and caspase-9 assays ^28^. We also examined the expression of two BCL-2 family proteins, BCL-2 and Bax, that may regulate the endogenous apoptotic pathway.

High expression of BCL-2 reduced apoptosis, whereas high expression of Bax promoted apoptosis ^29^. Ischemia/reperfusion stimulation induces translocation of Bcl-2 family proteins such as Bax to mitochondria and alters their outer membrane to release pro-apoptotic proteins such as cytochrome C. The abnormally increased cytochrome C binds to the cytoplasmic protein apaf1 to form apoptosomes and activate the caspase-9 and caspase-3 systems^30^. Upstream caspase-9 (promoter) activates downstream caspase-3 (effector). The results showed that I3C significantly reduced the expression level of caspase-9 in ischaemic brain tissue. We also observed a corresponding decrease in caspase-3 expression in ischemic brain tissue of I3C-pretreated rats. Analysis of BCL-2 and Bax proteins showed that I3C exerted anti-apoptotic effects by inhibiting the expression of Bax and increasing the expression of BCL-2. We also found that I3C treatment reduced neuronal loss after ischemia–reperfusion injury (Fig. 3).

Our study demonstrates that I3C treatment reduces M1-type microglia after cerebral ischemia–reperfusion injury, thereby antagonising early inflammation in ischemia–reperfusion injury, reducing neuronal cell loss, and improving neurological function in rats. In conclusion, I3C has a significant therapeutic effect on rat brain injury after cerebral ischaemia–reperfusion by modulating microglia. However, the present study only focused on the inflammatory response in the early stages of cerebral ischemia. Therefore, other relevant experiments are needed to assess the mechanisms by which I3C treatment modulates the role of microglia in cerebral ischaemia.

## Conclusion

Our results suggest that I3C treatment acts as an anti-inflammatory agent by modulating microglia, thus providing neuroprotection after ischemia–reperfusion injury.I3C may be a potential drug for the treatment of Ischemia/reperfusion injury in the brain. The anti-inflammatory function of I3C treatment may reduce apoptosis and ultimately promote recovery from CIRI.The molecular mechanisms of microglia modulation and anti-inflammatory protection by I3C need to be further investigated. Targeting apoptosis and inflammation with I3C may be a potential therapeutic strategy, but still needs to be tested in human studies.

## Materials and methods

### Materials

The I3C (#I-7256, Sigma-Aldrich, Munich, Germany) was dissolved in a solution containing dimethyl sulfoxide (DMSO), PEG300, Tween-80, and saline^31^.

### Animals

Six- to eight-week-old male SD rats (180–220 g) were obtained from the Experimental Animal Center of Nantong University. Rats were housed under standard laboratory conditions (5 rats per cage, 12 h light–dark cycle, 08:00 ~ 20:00 light on, 24 ± 1 °C ambient temperature, 55 ± 10% relative humidity) for 2 weeks with free access to water and food. Animal experiments were approved by the University Animal Ethics Committee of Nantong University (Permit Number: 2110836) and were conducted in accordance with the internationally accepted guidelines for the use of animals in toxicology adopted by the Society of Toxicology in 1999.

### Cell culture

Rat microglia (HAPI) were cultured in vitro in an incubator (5% CO2, 37 °C) containing 10% Australian origin fetal bovine serum (10100147, Gibco, USA), 1% penicillin/streptomycin and high sugar medium (10-013-CV, DMEM, Corning, NewYork, USA).

### Experimental design and drug treatment

After 2 weeks of habituation, 60 rats were randomly divided into sham-operated group, MCAO group and MCAO + I3C group, 20 rats in each group. I3C (150 mg/kg, once daily) was administered intraperitoneally to the rats on three consecutive days^32,33^, immediately after surgery on the first day, and at a fixed time of 12 am on the second and third days after surgery. I3C was formulated at the time of each daily administration and dissolved in Dimethylsulfoxide (DMSO).

HAPI cells of appropriate density were inoculated in 6-well plates containing complete culture medium and divided into blank group, OGD/R group and OGD/R + I3C group. There were at least 3 replicates in each group. I3C group HAPI cells were pretreated with 50 μM/mL I3C for 4 hours^19^.

### Establishment of the CIRI Model and OGD/R model

The procedure of the middle cerebral artery occlusion-reperfusion (MCAO/R) model is described below^34^. Rats were anesthetized by inhalation of isoflurane via the isoflurane delivery system. The rats were fixed in the supine position and the hair on the neck was shaved off with a razor. The rat's neck was disinfected with a cotton ball containing iodophor. Then a small incision of about 1 cm was made in the midline of the neck with surgical scissors to bluntly separate the subcutaneous tissue and muscle and expose the right common carotid artery (CCA), internal carotid artery (ICA) and external carotid artery (ECA). The distal common carotid artery (CCA) was ligated, and the internal carotid artery (ICA) was temporarily blocked with an arterial clip. The mice in the sham-operated group were subjected to the same procedure except for obstruction. The body temperature of the rats was maintained at 37 °C ± 0.5 °C, and the basic vital signs of the rats were closely monitored. Indicators were tested three days after surgery.

The OGD/R model was established as described below^35^. HAPI microglia were inoculated in sugar-free DMEM, divided into two groups, one of which was supplemented with I3C and transferred to an incubator at 37 °C, 94% N_2, 1% O_2 and 5% CO_2 for 4 hours^36^. The sugar-free DMEM was then replaced with normal medium (10% Australian-derived fetal bovine serum, 1% penicillin/streptomycin and high sugar medium) and incubated in an aerobic incubator for 24 h.

### Neurological assessment

The modified Neurological Severity Score(mNSS) consisted of motor, sensory, reflex and balance assessments with a normal score of 0 and a maximum score of 18. Each mouse was assessed on a score of 0 to 18 on days 1, 3, 7 and 14 after MCAO surgery.

### Water maze experiment

Trained rats were gently placed into the pool from each of the four quadrants facing the pool wall, the platforms were removed from the pool, and the rats were placed into the pool in the same position as the original platforms. The swimming path of the rats was then recorded for 2 min and tracked using computer software during the testing phase.

### Measurement of infarction volume

After 3 days of continuous supplementation with I3C, the rats were executed after deep anesthesia. The whole brain was quickly removed and placed in a Petri dish and dried after washing the remaining blood clots from the brain. The brain was placed in a brain tank, frozen at − 20 °C for 30 min, and cut into 2-mm coronal sections with a razor blade. The sections were immersed in 2% TTC dye for 30 min at 37 °C under dark conditions. Then fixed with 4% paraformaldehyde overnight. Uninjured brain tissues were stained red and brain infarction tissues were stained white. Finally, cerebral infarction volume was calculated using Image J software.

### Hematoxylin and eosin (H&E) staining

Paraffin sections were dewaxed and washed with different concentrations of ethanol and distilled water, then transparent with xylene. Then they were stained with H&E and sealed with neutral resin. Finally, they were observed under a microscope and photographed. The tissue sections are also scored. Scores are judged on the morphological structure of the cells and the condition of the pericellular area. 0 points: normal; 1 point: small amount of cell morphological alterations, no vacuoles around the cells; 2 point: Significant morphological changes with a few vacuoles around the cells; 3 points: significant morphological changes with significant vacuoles around the cells; 4 points: substantial morphological changes with a large number of vacuoles around the cells.

### Nissl staining

After dewaxing, the paraffin sections were washed three times with PBS buffer and then immersed in nylon staining solution at 60 °C for 30 min. The sections were again washed three times with PBS and dried at 60 °C. Then after xylene treatment, the sections were sealed with neutral glue. Finally, the pathology of neurons in the rat brain tissue was observed under a microscope and photographed. The number of neurites in each section was counted independently by three individuals in a triple-blind situation and the average was taken.

### Survival analysis

Sixty SD rats divided into three groups were used for a 7-day survival study. After surgery, the rats recovered in separate cages and were fed standard chow. Survival was assessed every 24 h after surgery until 7 days post-surgery. All animals in the treatment group were treated with the same dose of I3C (150 mg/kg/d).

### TUNEL assay

Apoptosis was detected using the TUNEL one-step apoptosis assay kit (Meilunbio, Dalian, China). Brain tissues were taken and fixed in 4% paraformaldehyde (PFA), dehydrated, and OCT-embedded. The OCT-embedded sections were cut into 10 μm-thick sections, then treated with paraformaldehyde, permeabilized with 0.5% Triton X-100, and incubated in the prepared working solution for 60 min at 37 °C according to the manufacturer's instructions. cells in six-well plates were fixed with 4% paraformaldehyde (PFA) and washed three times in PBS. Finally, the cells were analyzed under a fluorescence microscope (Leica, Wetzlar, Germany) and photographed.

### Cell viability/cytotoxicity detec

Three groups of cultured cells (blank group, OGD/R group OGD/R + I3C group) were inoculated into six-well plates, and the cells were washed two to three times with PBS according to the instructions (Meilunbio, China) to ensure that the active esterase contained in the culture medium was removed. 100 μl of dye solution was added to each well (working solution was added to 10 ml of PBS along with 5 μl of PI solution and 5 μl of calcein Am solution). Cells were observed and photographed by fluorescence microscopy after incubation at 37 °C for 30–60 min.

### CCK8 assay

Add 100 μl of configured cell suspension for each group to a 96-well plate. Pre-incubate the plates in an incubator for 24 h (37 °C, 5% CO2). Add 10 μl of CCK8 solution to each well and incubate the plate for 1–4 h in the incubator. Finally, the absorbance at 450 nm is measured by an enzyme marker.

### Enzyme-linked immunosorbent assay (ELISA)

IL-1β and IL-10 protein levels in each group of cells were quantified using ELISA kits according to the manufacturer's instructions.

### Apoptotic protein western blotting

Total proteins were extracted from ischaemic regions of brain tissue and protein concentrations were determined using a BCA kit. Protein samples were boiled for 10 min and then separated in 10% SDS-PAGE gels at 100 V for 100 min. It was then transferred to a PVDF membrane at 400 mA. The membranes were then blocked with 5% skimmed milk for 2 h at room temperature on a shaker, washed three times with TBST and incubated with primary antibody overnight on a shaker at 4 °C. The membranes were washed three times with TBST and incubated with secondary antibody for 1.5 h at room temperature. The membranes were developed using an ECL chemiluminescence instrument. The membranes were developed using an ECL chemiluminescence instrument and quantitatively analysed using ImageJ software.

### RNA extraction and real-time PCR

Total RNA was extracted using the Trizol kit according to the manufacturer's instructions and the RNA concentration was determined.

The reverse transcription reaction conditions were 42 °C, 15 min and 85 °C, 5 min. The mixed samples were added to wells of a 96-well plate and the polymerase chain reaction was carried out at 95 °C, 10 min denaturation, 95 °C, 15 s, 60 °C, 60 s, 40 cycles. The following primers were used in the experiment: IL-1β : 5′-tgccaccttttgacagtgatg-3′(f), 5′-tgatgtgctgcagatt-3′(r).

- IL-6:5′-tctggagcccaccaagaacgatag-3′(f), 5′-gtcaccagcatcagtcccaagaag′-(r).
- IL-4:5′-aaaactttgaacagcctacag-′(f),5′-ggtttccttctcagttgtgttc-3′(r).
- IL-10:5′-gttgttaaaggagtccttgctg-3′(f), 5′-ttcacagggaagaaatcgatga-3′-(r).
- Casp3:5′-CTGGACTGCGGTATTGAGACA-3′(f),
- 5′-CGGGTGCGGTAGAGAGTAAGC-3′(r)
- CASP9:5′-CCTTGTGTCCTACTCCACCTTCC-3′(f),
- 5′-GGAAGTTAAAACAGCCAGGAATC-3′(r)
- Bax:5′-GGGTGGTTGCCCTTTTCTACTT-3′(f),
- 5′- GAAGTCCAGTGTCCAGCCCAT-3′(r)
- BCL-2:5′-TTGTGGCCTTCTTTGAGTTCG-3′(f),
- 5′-GCATCCCAGCCTCCGTTAT-3′(r)
- INOS:5′-CTACTACTACCAGATCGAGCCCTG-3′(f),
- 5′-CTAGCGCTTCCGACTTTCCT-3′(r)

### Immunofluorescence staining

After the brain tissue samples were frozen and sectioned, the frozen sections were dried in an oven at 37 °C for 1 h. The sections were washed three times with PBS solution for 10 min each time. Then, the sections were soaked in antigen repair solution for 30 min and washed three times with PBS solution. Sections were blocked with goat serum for 60 min for antigen, and the blocking solution was removed. The appropriate amount of primary antibody was added and incubated overnight at 4 °C before removing the blocking solution, adding the appropriate amount of primary antibody and incubating at 4 °C for 16 h. Then, the secondary antibody was added and incubated for 2 h. The nuclei were stained with DAPI. Finally, the cells were observed under fluorescence microscope and photographed.

### Statistical analysis

One-way analysis of variance (ANOVA) or two-way ANOVA was used for multiple comparisons, and all data were statistically analyzed using GraphPad Prism9 software. Data were expressed as mean ± standard deviation of the mean (SEM). Values of P < 0.05 were considered statistically significant.

### Ethics statement

The animal study was reviewed and approved by the University Animal Ethics Committee of Nantong University (Permit Number: S20221212-004). The experiment was conducted in accordance with ARRIVE guidelines.

### Supplementary Information

Supplementary Information.

## Data Availability

The datasets used and/or analyzed during the current are study available from the corresponding author upon reasonable request.
