# Supplementary material for: Indole-3-carbinol (I3C) reduces apoptosis and improves neurological function after cerebral ischemia–reperfusion injury by modulating microglia inflammation
Source: Sci Rep. 2024 Feb 7;14:3145. doi: 10.1038/s41598-024-53636-6 (PMC10850550; doi:10.1038/s41598-024-53636-6)
Supplement: Supplementary file 1 — Supplementary Information. [file 41598_2024_53636_MOESM1_ESM.pdf]

Supplementary

Supplementary Figure S1

(A)Representative maps of fluorescence co-localisation of the inflammatory factor INOS and the microglia marker IBA1. (B)Quantitative analysis of the mean fluorescence intensity of INOS. P-values were calculated using one-way· ANOVA. n.s.P≥0.05,*P<0.05,**P<0.01,.***P<0.001,****P<0.0001.

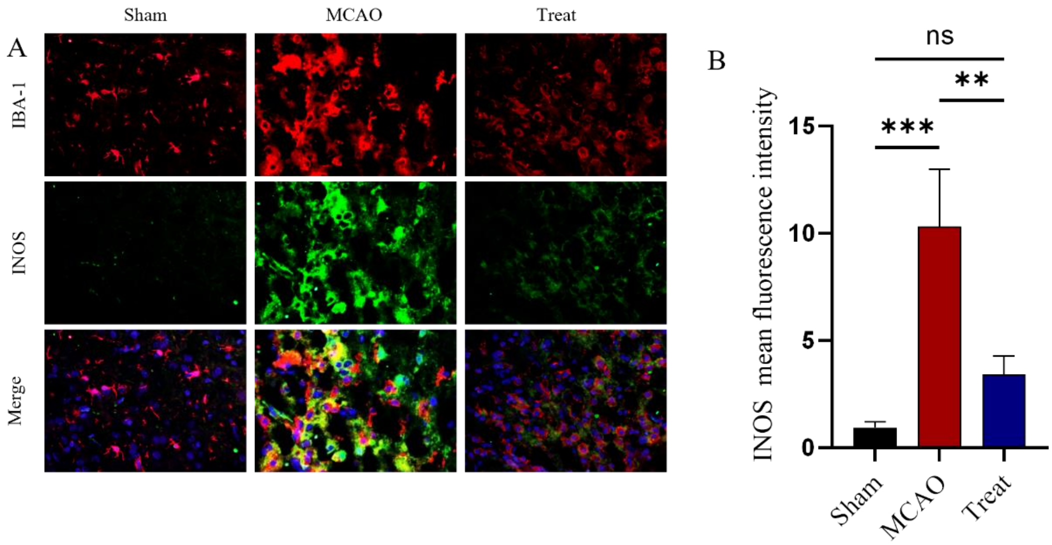

Supplementary Figure S2.

Quantitative statistics of cell survival in microglia CCK8 experiments. P-values w ere calculated using one-way· ANOVA. n.s.P≥0.05,*P<0.05,**P<0.01,.***P<0.001, ****P<0.0001.

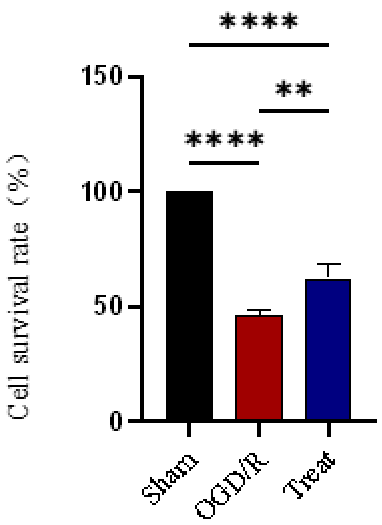

Supplementary Table S1:

The total mNSS score was 18. The behavioral ability was evaluated according to different scores. Score: 13-18, severe; 7-12, moderate; 1-6, mild injury. The detailed rules are shown in **Supplementary Table S1**.

mNSS			score
**exercise test**	Tail test	The forelimb flexion	1
		The hind limb flexion	1
		The head deviated from the vertical axis> 100 in 30s	1
	Place rat on the floor (normal =0; max =3)	Normal walking	0
		Can't walk straight	1
		Turn the circle to the paraplegic side	2
		Dumping to the paraplegic side	3
**sensory testing**	Placement trials (visual and tactile test)		1
	Proprioception test (deep sensation, pressing the paw at		1

	the edge of the table to stimulate limb muscles)	
**Balance beam test (normal value =0; max. value =6)**	Stable balance posture	0
	Grasp the edge of the balance beam	1
	Hold the balance beam tightly, and one body falls from the balance beam	2
	Hold the balance beam tightly, and the two limbs fall from the balance beam or rotate on the balance beam (> 60 seconds)	3
	Tried to balance on the balance beam but dropped (> 40 seconds)	4
	Tried to balance on the balance beam but dropped (> 20 seconds)	5
	Drop; not attempt to balance on balance beam (<20 seconds)	6
**Loss of the reflex and abnormal movement**	Eicle reflex (shake head when touching the external auditory canal)	1
	Corneal reflex (blink when touching the cornea with cotton silk)	1
	Panic reflex (motor response to noise from blast cardboard)	1
	Epilepsy, myoclonus, and dystonia	1

Western blot original image

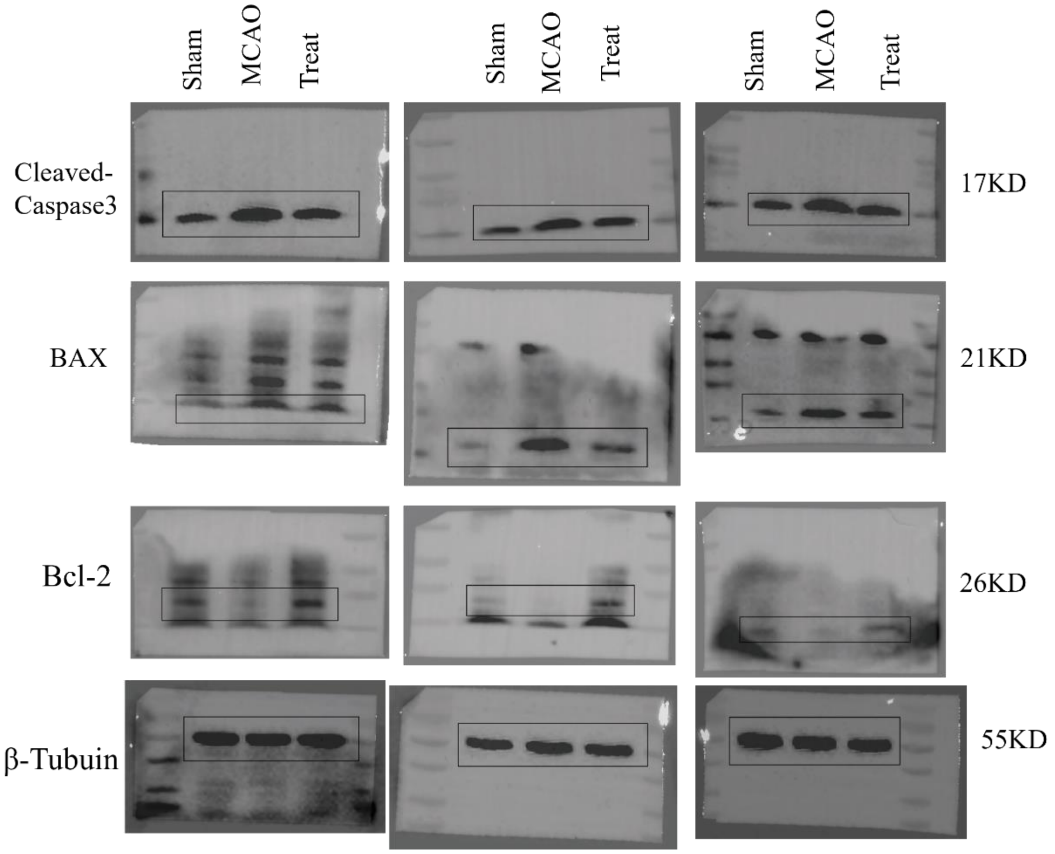

We have clipped the PVDF membrane before transferring the protein gel to the PVDF membrane, so this is already our Western blot original image.
